# A karyosystematic analysis of some water beetles related to *Deronectes* Sharp (Coleoptera, Dytiscidae)

**DOI:** 10.3897/CompCytogen.v5i3.1185

**Published:** 2011-08-24

**Authors:** R.B. Angus, A.G. Tatton

**Affiliations:** 1 Department of Entomology, The Natural History Museum, Cromwell Road, London SW7 5BD UK; 2 School of Biological Sciences, Royal Holloway, University of London, Egham, Surrey TW20 0EX, UK

**Keywords:** Chromosomes, karyotypes, Dytiscidae, *Deronectes*, *Stictotarsus*, *Scarodytes*, *Trichonectes*, *Nebrioporus*, species, phylogeny

## Abstract

An account is given of the karyotypes of five species and one additional subspecies of *Deronectes* Sharp, 1882, three species of *Stictotarsus* Zimmermann, 1919, one species of *Trichonectes* Guignot, 1941, four species of *Scarodytes* Gozis, 1914 and 17 species of *Nebrioporus* Régimbart, 1906. *Deronectes* species are characterised by a neo-Xy system of sex chromosomes and autosome numbers ranging from 60 (*Deronectes ferrugineus* Fery et Brancucci, 1987 and *Deronectes wewalkai* Fery et Fresneda, 1988) through 48 (*Deronectes latus* (Stephens, 1829), *Deronectes angusi* Fery et Brancucci, 1990) to 28 (*Deronectes costipennis* Brancucci, 1983, *Deronectes costipennis gignouxi* Fery et Brancucci, 1989 and *Deronectes platynotus* (Germar, 1834)). The three species of *Stictotarsus*, *Stictotarsus duodecimpustulatus* (Fabricius, 1792), *Stictotarsus procerus* (Aubé, 1838) and *Stictotarsus bertrandi* (Legros, 1956), all belonging to the *Stictotarsus duodecimpustulatus* group of species, have karyotypes comprising 54 autosomes and neo-Xy sex chromosomes. *Trichonectes otini* Guignot, 1941 has 48 autosomes and an X0 system of sex chromosomes, an arrangement shared with the 17 species of *Nebrioporus* Régimbart. The four *Scarodytes* species, *Scarodytes halensis* (Fabricius, 1787), *Scarodytes nigriventris* (Zimmermann, 1919), *Scarodytes fuscitarsis* (Aubé, 1838) and *Scarodytes malicky*i Wewalka, 1997, all have 54 autosomes and X0 sex chromosomes. The karyotypes of the various species are found to be distinctive and to support separation of these species from one another. In two cases (*Nebrioporus martinii* (Fairmaire, 1858) and *Nebrioporus sardus* (Gemminger et Harold, 1868), and *Scarodytes halensis* and *Scarodytes fuscitarsis*) the karyotypes require the recognition of the taxa as full species, not subspecies. The implications of these data for the generic classification are considered. The data are found to be compatible with the DNA-based phylogeny proposed by Ribera (2003), where the enlarged *Stictotarsus* proposed by Nilsson and Angus (1992) is found to be unsatisfactory.

## Introduction

The *Deronectes* group of genera comprise small diving beetles (Dytiscidae) which belong to the subfamily Hydroporinae, tribe Hydroporini. They typically inhabit stony or gravelly rivers or lakes, and often have a characteristic, quite flattened appearance. Beyond that, it is not easy to formulate a set of characteristics which delimit the group, and in a number of cases the scope and arrangement of the included genera are not clear. Nilsson and Angus (1992) sought to clarify the situation, and in particular to separate the mainly northern hemisphere *Deronectes* Sharp, 1882 group from various superficially similar southern hemisphere genera. They separated *Deronectes* itself as having a more or less uniformly coloured upper surface, parameres without an apical hook, and larvae without extra swimming-hairs on the legs. The other genera, all of whose larvae have extra swimming-hairs on the legs and whose upper surfaces are patterned, were divided into those whose parameres had an apical hook and those without such a hook. The species that possessed hooked parameres were placed in the genera *Nebrioporus* Regimbart, 1906 and *Scarodytes* Gozis, 1914, while those without them were placed in an expanded *Stictotarsus* Zimmermann, 1919. This expanded *Stictotarsus* group has not been favourably received. The genus *Trichonectes* Guignot, 1941, which was included in *Stictotarsus* by Nilsson and Angus, was reinstated by Ribera (2003) because DNA analysis showed it to be only distantly related to other *Stictotarsus*, and Angus (2010b) erected the genus *Boreonectes* for the *Stictotarsus griseostriatus* group of Nilsson and Angus.

Nilsson and Angus reported some chromosomal data, which indicated a considerable diversity of chromosome number and sex-determining mechanisms within the group, with the only notable generic uniformity being shown by *Nebrioporus*, whose studied species all had 2n = 48 + X0 (♂), XX (♀). So far karyotypes have been published for six species of *Nebrioporus* – *Nebrioporus carinatus* (Aubé, 1838), *Nebrioporus fabressei* (Régimbart, 1901) and *Nebrioporus croceus* Angus, Fresneda et Fery, 1992, all Spanish species (Angus et al. 1992); and three Egyptian species, *Nebrioporus crotchi* (Preudhomme de Borre, 1871), *Nebrioporus insignis* (Klug, 1833) and *Nebrioporus lanceolatus* (Walker, 1871) (Saleh Ahmed et al. 2000).

Of the other genera, *Scarodytes halensis* (Fabricius, 1787) was reported to have 27 pairs of autosomes and an X0 sex chromosome system, while three *Stictotarsus* (*duodecimpustulatus* group) species have 27 pairs of autosomes and a neo-XY sex chromosome system (*Stictotarsus duodecimpustulatus* (Fabricius, 1792), *Stictotarsus procerus* (Aubé, 1838) and *Stictotarsus bertrandi* (Legros, 1956)). *Boreonectes* species have chromosome numbers ranging from 50–60 autosomes and an X0 sex chromosome system (Dutton and Angus 2007; Angus 2008, 2010a, b). The remaining genus for which some chromosome data have been reported is *Deronectes*. Here autosome numbers ranging from 14 to 30 pairs have been observed, with a neo-XY sex chromosome system in all cases. Considerable variation in the sizes of both autosomes and sex chromosomes was also noted.

The main aims of the present work are to present karyotypes from species of these genera for which such information is not yet available, and to see to what extent, if any, these other genera show generic patterns of karyotype, such as that shown by *Nebrioporus*.

The chromosomal features available for analysis are limited to their size, given as the length of each chromosome expressed as a percentage of the total haploid autosome length in the nucleus (relative chromosome length (RCL)) – which compensates for differing degrees of chromosome condensation in different preparations, the centromere position, given as the percentage of the total length of a chromosome occupied by its shorter arm (centromere index (CI)), heterochromatin development and distribution (indicated by C-banding, in this case only available for *Stictotarsus*), and any obvious secondary constrictions on the chromosomes. Detailed G-banding, which allows accurate identification of individual chromosome arms or even portions of arms, in mammals, is not possible with insects, and no attempt was made to use DNA probes on these nuclei. The centromere indices of the chromosomes are most conveniently expressed as the standard terms for centromere position. Based on Sumner (2003), these are: metacentric (median centromere), CI 50–46; submetacentric (centromere clearly not quite median), CI 45–26; subacrocentric (centromere almost at one end of the chromosome), CI 25–16; acrocentric (including telocentric), CI < 16.

One result of the limited range of features available is that in some of these karyotypes, particularly those involving fairly large numbers of chromosomes similar in size and centromere position, there is inevitably a degree of ambiguity. However, even when comparison is limited to clearly expressed differences, many of the species are seen to have distinctive karyotypes.

## Material and methods

The material used in this study is archive photographs of mitotic chromosomes of various species, accumulated by R.B. Angus. The details – species, localities, collector and date, and number of specimens analysed – are given in Table 1. These preparations have been accumulated over more than 20 years, and in many cases only a few successful preparations were obtained, even though large numbers of beetles were used in attempts. The number analysed given in the table refers to the number of beetles from which successful preparations were obtained.

**Figure 1.**
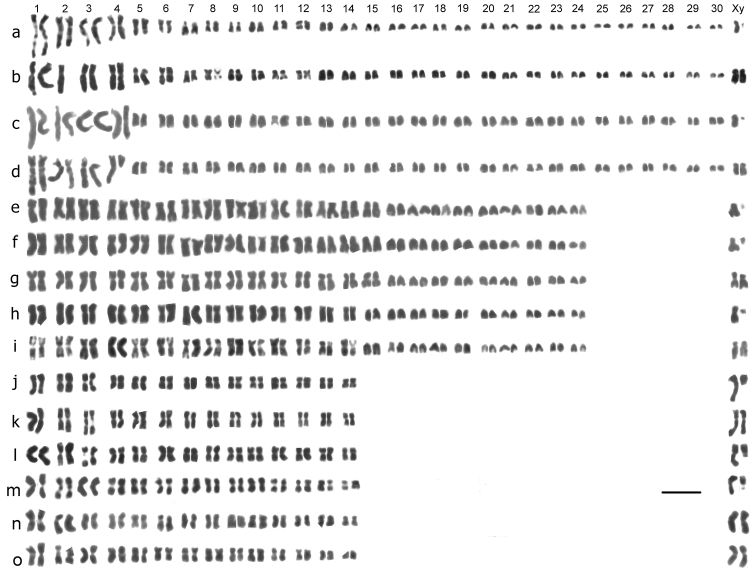
Mitotic chromosomes from mid-gut cells of *Deronectes* species, arranged as karyotypes. **a** *Deronectes ferrugineus*, ♂ **b** *Deronectes ferrugineus*, ♀ **c** *Deronectes wewalkai*, ♂ **d** *Deronectes wewalkai*, ♀ **e, f** *Deronectes latus*, ♂ **g** *Deronectes latus*, ♀ **h** *Deronectes angusi*, ♂ **i** *Deronectes angusi*, ♀ **j** *Deronectes costipennis costipennis*, ♂ **k** *Deronectes costipennis costipennis*, ♀ **l** *Deronectes costipennis gignouxi*, ♂, **m** *Deronectes platynotus*, ♂, Bulgaria **n, o** *Deronectes platynotus*, ♀, Germany. Bar = 5µm.

The methods of chromosome preparation are outlined by Dutton and Angus (2007). Photographs were printed at a magnification of X 3000. Chromosomes were cut from the photographs, paired up and plated as karyotypes. They were then scanned into a computer for analysis using Adobe Photoshop. This facilitated necessary rearrangement of various karyotypes, as well as bringing them together on single plates, for comparison of species and populations. In addition to this, the photographs were “cleaned up” by adjustment of contrast and brightness (to allow for different backgrounds), and extraneous blemishes were removed.

The chromosomes were measured, so that the relative chromosome length (RCL) and centromere index (CI) of the chromosomes could be calculated. Because of the scarcity of replicates no statistical analysis has been attempted with these data, and the RCL and CI values are given as a rough guide only.

**Table 1.** Material analysed.

*Species*	*Locality*	*Collector, date*	*Number analysed*
*Deronectes ferrugineus* Fery et Brancucci	Portugal, Distr. Guarda, Serra do Estrela	H. Fery 1990	2 ♂ 1 ♀
*Deronectes wewalkai* Fery et Fresneda	Spain, Provincia de Avila, Sierra de Gredos	H. Fery 1990	2 ♂ 1 ♀
*Deronectes latus* (Stephens)	England, Hampshire, New Forest	R.B. Angus 1990	3 ♂ 1 ♀
*Deronectes angusi* Fery et Brancucci	Spain, Provincia de Burgos, Sierra de Arlanzon	H. Fery 1990	1 ♂ 1 ♀
*Deronectes costipennis costipennis* Brancucci	Portugal, Distr. Guarda, Serra do Estrela	H. Fery 1990	2 ♂ 1 ♀
*Deronectes costipennis gignouxi* Fery et Brancucci	Spain, Provincia de Leon, Posada de Valdeon	H. Fery 1990	4 ♂♂
*Deronectes platynotus* (Germar)	Bulgaria, Pindus Mountains	D. Bilton, 2006	1 ♂
	Germany, Saxonia, Weisseritz Kreis	L. Hendrich 2006	2 ♀♀
*Stictotarsus duodecimpustulatus* (Fabricius)	Scotland, Kirkcudbrightshire Clatteringshaws Loch	G.N. Foster 1990	1 ♂
	England, Hampshire, New Forest	R.B. Angus 1990	1 ♀
	Spain, Provincia de La Coruña, Esclavitud	R.B. Angus 1990	1 ♂ 1 ♀
	Spain, Provincia de Caceres, Abadia	R.B. Angus 1990	1 ♀
*Stictotarsus procerus* (Aubé)	Corsica, Haute Corse, Solenzara	R.B. Angus 1993	3 ♂ 1 ♀
	Corsica, Haute Corse, R.Casaluna, Pont de Lano	R.B. Angus 1993	2 ♂♂ 1 ♀
	Sardinia, Provincia de Nuoro, Posada	R.B. Angus 1994	2 ♂ 1 ♀
*Stictotarsus bertrandi* (Legros)	Spain, Provincia de Lugo. Rio Landro	J. Diaz Pazos 1990	1 ♂ 1 ♀
*Trichonectes otini* (Guignot)	Spain, Provincia de Cordoba, Salinas de la Maturra, Baena	M. Baena 1993	4 ♂♂
	Spain, Provincia de Sevilla, S of Osuna	H. Fery 1993	2 ♂ 1 ♀
*Scarodytes halensis* (Fabricius)	England, Oxfordshire, Stanton Harcourt	R.B. Angus 1990	2 ♂♂ 1 ♀
	France, Alpes Maritimes, Menton	H. Fery 1990	1 ♀
*Scarodytes nigriventris* (Zimmermann)	Corsica, Haute Corse, Solenzara	R.B. Angus 1993	1 ♂
	Corsica, Haute Corse, R.Casaluna, Pont de Lano	R.B. Angus 1993	1 ♂ 1 ♀
*Scarodytes fuscitarsis* (Aubé)	Sardinia, Provincia de Nuoro, Budoni	R.B. Angus 1994	3 ♂ 1 ♀
*Scarodytes malickyi* Wewalka	Crete, Nomos Rethymnou. Spili – Gerakari	R.B. Angus 1996	2 ♂♂
*Nebrioporus ceresyi* (Aubé)	Cyprus, Akrotiri, Zakaki marshes	R.B. Angus 1995	2 ♂♂
	Sardinia, Provincia di Oristano, Sinis Peninsula	R.B. Angus 1994	4 ♂♂
*Nebrioporus baeticus* (Schaum)	Spain, Provincia de Sevilla, S of Osuna	H. Fery 1993	2 ♂♂
	Provincia de Cordoba, Castro del Rio	M. Baena 1993	1 ♂
*Nebrioporus canaliculatus* (Lacordaire)	Spain, Provincia de Burgos, 4 km SSW of Sasamon	H. Fery 1994	1 ♂
	Sweden	A.N. Nilsson, 1991	1 ♂
*Nebrioporus bucheti* (Régimbart)	France, Alpes Maritimes, Menton	H. Fery 1990	1 ♂, 1 ♀
*Nebrioporus martinii* (Fairmaire)	Corsica, Haute-Corse, Solenzara	R.B. Angus 1993	2 ♂♂
*Nebrioporus sardus* (Gemminger et Harold)	Sardinia, Provincia di Nuoro, Budoni	R.B. Angus 1994	1 ♂
*Nebrioporus depressus* (F.)	England, Cumbria, Talkin Tarn	R.B. Angus 1987	1 ♂
*Nebrioporus depressus- elegans* intermediate	Scotland, Kirkcudbrightshire, Clatteringshaws Loch	G.N. Foster 1987	2 ♂♂
*Nebrioporus elegans* (Panzer)	England, Oxfordshire, Cassington	R.B. Angus 1987	2 ♂♂
*Nebrioporus assimilis* (Paykull)	England, Cumbria, Grasmere	R.B. Angus 1987, 1990	3♂♂
*Nebrioporus carinatus* (Aubé)	Spain, Provincias de La Coruña, Lugo & Palencia	(Angus et al. 1992)	3 ♂♂, 1 ♀
*Nebrioporus fabressei* (Régimbart)	France, Pyrenées Orientales; Spain, Provincia de Segovia	(Angus et al. 1992)	4 ♂♂
*Nebrioporus croceus* Angus, Fery et Fresneda	Spain, Provincia de Soria.	(Angus et al. 1992)	5 ♂♂
*Nebrioporus amicorum* Toledo	Crete, Nomos Rethymnou. Spili – Gerakari	R. B. Angus 1996	1 ♂
*Nebrioporus lanceolatus* (Walker)	Egypt, Sinai, St Katherine & El Gharandal	(Saleh Ahmed et al. 2000)	3 ♂♂
*Nebrioporus insignis* (Klug)	Egypt, Sinai, St Katherine	(Saleh Ahmed et al. 2000)	1 ♂
*Nebrioporus crotchi* (Preudhomme de Borre)	Egypt, Sinai, St Katherine	(Saleh Ahmed et al. 2000)	2 ♂♂
*Nebrioporus canariensis* (Bedel)	Canary Islands, Tenerife	A.N. Nilsson 1991	2 ♂♂

## Results

### DeronectesSharp, 1882

Fig. 1, a – o, shows mitotic chromosomes from mid-gut cells, of the six *Deronectes* species (and one subspecies) studied. The diploid numbers of autosomes range from 60 (*Deronectes ferrugineus* and *Deronectes wewalkai*) through 48 (*Deronectes latus* and *Deronectes angusi*) to 28 (*Deronectes costipennis*, *Deronectes costipennis gignouxi* and *Deronectes platynotus*). The sex chromosomes are XY or Xy (♂), XX (♀). In *Deronectes ferrugineus*, *Deronectes wewalkai*, *Deronectes latus* and *Deronectes angusi*, the y-chromosomes are dot-like, but in *Deronectes costipennis*, *Deronectes costipennis gignouxi* and *Deronectes platynotus* the Y chromosomes are rather larger, and match the short arms of the X chromosome, strongly suggesting that this is a neo-XY system. The features of the various species are discussed below.

**Figure 2.**
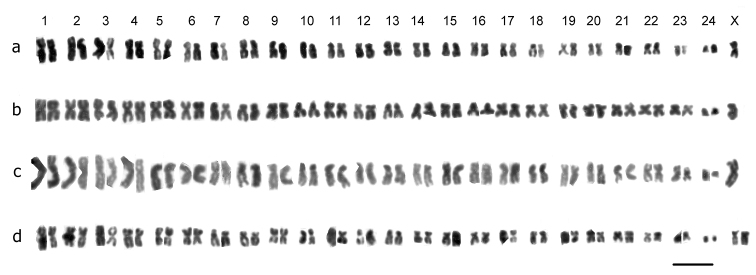
Mitotic chromosomes from mid-gut cells of*Trichonectes otini*, arranged as karyotypes. **a** ♂, Cordoba **b, c** ♂♂, Sevilla **d** ♀, Sevilla. Bar = 5µm.

*Deronectes ferrugineus* Fery et Brancucci, 1987. Fig. 1 a (♂), Fig. 1 b (♀). 2 n = 60 + Xy (♂), XX (♀). Pair 1 is clearly the longest (RCL about 14), with pairs 2–4 only slightly smaller (RCL about 6.5–10) placed together as they cannot be distinguished from one another with certainty). These four pairs of chromosomes have the long arm much longer than the short arm (CI about 30–40), and although no C-banding is available, the way the chromatids of these long arms lie closely applied to another suggests that they are largely heterochromatic (comparing the heterochromatic long arms of some of the *Stictotarsus* chromosomes shown in Fig. 2). Pairs 5 and 6 are about half the length of pairs 2–4 (RCL about 4.5), and are almost metacentric. Pairs 7–12 show a gradual decrease in RCL, from about 4 to about 3. Pairs 7, 9 and 10 are subacrocentric, while pairs 8, 11 and 12 are submetacentric. Pairs 13–30 are acrocentric and their RCLs range from 2.5–0.9. The X chromosome (RCL about 2.6) is submetacentric, similar in length to autosomes 5 and 6, but appearing a little more dense. The y chromosome is dot-like, thus is too small for the RCL to be measured.

*Deronectes wewalkai* Fery et Fresneda, 1988. Fig. 1 c (♂), Fig. 1 d (♀). 2 n = 60 + Xy (♂), XX (♀). The karyotype of this species shows no detectable difference from that of *Deronectes ferrugineus*. One peculiar feature of the female karyotype is the loss of the long arm of one replicate of autosome 4. This appears to be a one-off deletion from this nucleus, but as this was the only platable nucleus obtained from this beetle it is impossible to say whether other cells showed the same deletion. If these long arms are heterochromatic, as suggested for *Deronectes ferrugineus*, the genetic consequences of the deletion would be minimal.

*Deronectes latus* (Stephens, 1829). Fig. 1 e, f (♂), Fig. 1 g (♀). 2n = 48 + Xy (♂), XX (♀). Autosome pairs 1–13 are all about the same size (RCLs range from about 6 to about 5), and are all more or less metacentric. Pairs 14–16 are slightly smaller, RCL about 4.5, and are subacrocentric. Pair 17 is acrocentric and markedly smaller than pair 16 (RCL about 3), and pairs 17–25 are all similar in shape, with a gradual decrease in RCL from 3 to 2.3. The X chromosome is subacrocentric, and similar in size to autosome 16. The y chromosome is dot-like.

*Deronectes angusi* Fery et Brancucci, 1990. Fig. 1 h (♂), Fig. 1 i (♀). 2n = 48 + Xy (♂), XX (♀). The karyotype of this species is very similar to that of *Deronectes latus*, with the exception of autosome 16, which is similar to autosome 17, unlike that of *Deronectes latus*, which is similar to autosome 15. This species was originally considered as a somewhat peculiar Spanish form of *Deronectes latus*, but the discovery of the chromosomal difference convinced H. Fery (pers. comm.)that it was in fact a good species.

*Deronectes costipennis costipennis* Brancucci, 1983. Fig. 1 j (♂), Fig. 1 k (♀). 2n = 28 + XY (♂), XX (♀). Autosome pairs 1–3 are conspicuously large, RCLs about 12.5–8.5. Pair 2 is metacentric, while pairs 1 and 3 are submetacentric, towards the subacrocentric end of the range. Pairs 4–14 are more less metacentric and their RCLs range from about 7.8–3, with a gradual size decrease along the row. The X chromosome is large, RCL about 14.8, and submetacentric, CI about 30. The y chromosome, RCL about 5.5, is acrocentric and matches the short arm of the X chromosome – a typical neo-XY configuration.

*Deronectes costipennis gignouxi* Fery et Brancucci, 1989. Fig. 1 l (♂). 2n = 28 + XY (♂), XX (♀). This karyotype is almost certainly indistinguishable from that of *Deronectes costipennis costipennis*. The X chromosome (RCL) appears slightly shorter, but all the chromosomes appear more condensed than in the *costipennis* nuclei, and this is probably sufficient to account for the apparent difference. In any event, a lot more material would be needed to demonstrate so small a difference.

*Deronectes platynotus* (Germar, 1834).Fig. 1 m (♂), Fig. 1 n, o (♀). 2n = 28 + XY (♂), XX (♀). The karyotype appears very similar to that of *Deronectes costipennis*, but chromosome 1 is more metacentric and chromosome 2 has the distinct secondary constriction in this species, as against chromosome 3 in *Deronectes costipennis*. Although the RCLs of various chromosomes can be affected by irregularities of condensation, this difference appears consistent. It is worth mentioning that the Bulgarian male has the pointed aedeagus characteristic of *Deronectes platynotus platynotus*, not the truncated one found in the Greek *Deronectes platynotus mazzoldii*.

### TrichonectesGuignot, 1941

*Trichonectes otini* (Guignot, 1941). Fig. 2 a – c (♂), Fig. 2 d (♀). 2n = 48 + X0 (♂), XX (♀).The RCLs of the autosomes decrease rather evenly from about 6.5–2, and most of the autosomes are metacentric or submetacentric apart from pairs 7, 8, 10, 14–18, which are more or less subacrocentric and pair 24 which is acrocentric and, although small, not dot-like. The short arms of pairs 7, 10 and 12 appear to have secondary constrictions. The X chromosome is submetacentric, with a RCL of about 5.

### NebrioporusRégimbart, 1906

Chromosome data are now available for 17 species out of the 57 listed by Toledo (2009), with this number increased to 58 in the present work. The species are arranged according to the groupings suggested by Toledo (2009). In all cases the diploid number is 48 + X0 (♂), XX (♀).

**The** N. ceresyi **group**

**Figure 3.**
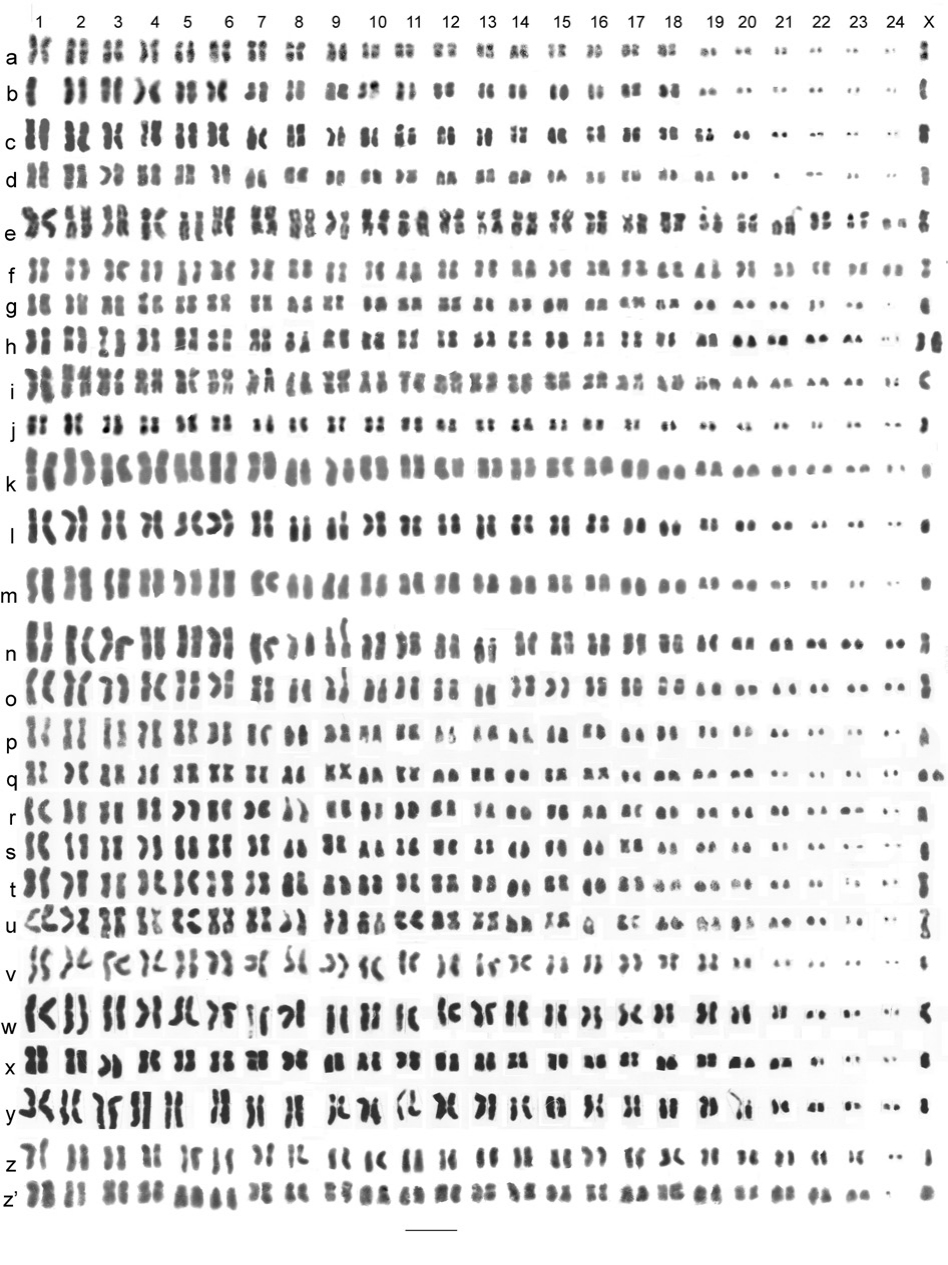
Mitotic chromosomes of *Nebrioporus* species, arranged as karyotypes. **a, b** *Nebrioporus ceresyi*, ♂, mid-gut, **a** Cyprus **b** Sardinia; **c, d** *Nebrioporus baeticus*, ♂, mid-gut, **c** Castro del Rio **d** S of Osuna; **e, f** *Nebrioporus canaliculatus*, ♂, mid-gut; **e** Sasamon **f** Sweden; **g, h** *Nebrioporus bucheti*, mid-gut, Menton, **g** ♂ **h** ♀; **i** *Nebrioporus martinii*, ♂, mid-gut, Corsica **j** *Nebrioporus sardus*, ♂, mid-gut, Sardinia **k** *Nebrioporus depressus*, ♂, mid-gut, Talkin Tarn **l** *Nebrioporus depressus-elegans* intermediate, ♂, mid-gut, Clatteringshaws Loch **m** *Nebrioporus elegans*, ♂, mid-gut, Cassington **n, o** *Nebrioporus assimilis*, ♂, mid-gut, Grasmere **p, q** *Nebrioporus carinatus*, mid-gut **p** ♂ **q** ♀; **r, s** *Nebrioporus fabressei*, ♂, testis **r** France **s** Spain; **t, u** *Nebrioporus croceus*, ♂ paratypes **t** testis **u** mid-gut **v** *Nebrioporus amicorum*, ♂, mid-gut, Crete **w** *Nebrioporus lanceolatus*, ♂, testis, St Katherine **x** *Nebrioporus insignis*, ♂, mid-gut, St Katherine **y** *Nebrioporus crotchi*, ♂, mid-gut, St Katherine **z, z’** *Nebrioporus canariensis*, ♂, mid-gut, Bar = 5 µm.

*Nebrioporus ceresyi* (Aubé 1838). Fig. 3 a, b (♂). The RCLs of autosome pairs 1–18 decrease fairly evenly from about 8 to 3.5. Pairs 19–21 have RCLs of about 2.5 while pairs 22–24 are almost dot-like, RCLs about 1.5. The X chromosome is metacentric, RCL about 5. Most of the autosomes are metacentric, with pairs 4, 7, 9, 11, 13, 14, 16, 17 and 19 more or less subacrocentric, some probably with secondary constrictions, though these cannot be seen in the preparations available.

*Nebrioporus baeticus* (Schaum, 1864). Fig. 3 c, d (♂). The karyotype is very similar to that of *Nebrioporus ceresyi*, but with some clear differences. The larger autosomes, in this case pairs 1–19, have RCLs decreasing evenly from about 8 to 3, with an abrupt decrease to pair 20, RCL about 2. Pairs 21–24 are all very small, RCLs about 1.5–1. The X chromosome is metacentric, RCL about 4. Most of the autosomes are more or less metacentric, with the obvious subacrocentrics being pairs 7, 9–13, 15–17 and 19.

**The** N. canaliculatus **group**

*Nebrioporus canaliculatus* (Lacordaire, 1835). Fig. 3 e, f (♂). The RCLs of the autosomes decrease evenly from about 5.5–2.2, with no dot-like chromosomes. The X chromosome is metacentric, RCL about 4. Most of the autosomes are more or less metacentric, with pairs 5, 9, 11, 14, 17, 19 and 21 either submetacentric or subacrocentric, and pairs 19 and 21 with secondary constrictions (Fig. 3 e).

**The** N. sansii **group**

*Nebrioporus bucheti* (Regimbart, 1898). Fig. 3 g (♂), h (♀). The RCLs of autosome pairs 1–23 decrease evenly from about 7.5–1.9. The RCL of pair 24 is about 1.6 and this pair can appear dot-like. The X chromosome is submetacentric, RCL about 4.6. Most autosomes are either metacentric or submetacentric, with pairs 7, 8, 10, 18–23 the most obvious subacrocentrics. The material belongs to the nominate subspecies, *Nebrioporus bucheti bucheti*.

*Nebrioporus martinii* (Fairmaire, 1858). Fig. 3 i (♂). The RCLs of the autosomes decreasing rather evenly from about 7.5–1.8. The X chromosome is metacentric, RCL about 4.5, similar to autosomes 11–12. Autosomes 1, 3, 10, 11, 12, 14 and 17 are clearly submetacentric and pairs 7, 8 and 18–24 are clearly subacrocentric, with the others more or less metacentric.

*Nebrioporus sardus* (Gemminger et Harold, 1868), stat. n. Fig. 3 j (♂). A more condensed preparation than the *Nebrioporus martinii* shown in Fig. 3 i. The RCLs of the autosomes decrease rather evenly from about 6–2, and the X chromosome is metacentric, RCL about 5, comparable with autosome 6. Autosomes 1, 3, 7, 8 , 12, 14, 15 and16 are clearly submetacentric, pairs 18–24 appear acrocentric to subacrocentric, and the rest are metacentric. The chromosome array presented here, although condensed, shows a number of clear differences from that of *Nebrioporus martinii*. Thus pair 8 is submetacentric in *Nebrioporus sardus* but almost acrocentric in *Nebrioporus martinii*, and pairs 10 and 11 are clearly metacentric in *Nebrioporus sardus* but submetacentric in *Nebrioporus martinii*. These differences cannot be resolved by slight rearrangement of the karyotype of either species, and strongly suggest that there has been chromosomal rearrangement since the two taxa separated. For this reason *Nebrioporus sardus* is here elevated to species rank.

**The** *Nebrioporus depressus* **group**

*Nebrioporus depressus* (Fabricius, 1775) and *Nebrioporus elegans* (Panzer, 1794). Fig. 3 k, l, m (♂). *Nebrioporus depressus* and *Nebrioporus elegans* are listed as separate species of a *Nebrioporus depressus* complex by Toledo, and as the regional occurrence of intermediate specimens is well documented (Balfour-Browne 1919, 1934; Franck 1935; Shirt 1983) they are considered together. Fig. 3 k is a good *Nebrioporus depressus*, Fig. 3 l is a Scottish intermediate specimen, and Fig. 3 m is an English *Nebrioporus elegans*. The three karyotypes show no differences from one another. The RCLs of autosome pairs 1–21 decrease evenly from about 7.5–2.2, while the three smallest pairs verge on dot-like and are thus more or less impossible to measure. The X chromosome is a small acrocentric, RCL about 2.8. Autosomes 8, 9 and 18–23 are subacrocentric to acrocentric, and the remainder are metacentric to submetacentric.

*Nebrioporus assimilis* (Paykull, 1798). Fig. 3 n, o (♂). The RCLs of the autosomes decrease evenly from about 6.5–1.8, with no dot-like autosomes. The X chromosome is metacentric, RCL about 4, comparable with autosome 11. Autosomes 8–12, and 18–24 are subacrocentric to acrocentric, and pairs 3, 7, 11, 12 and 14–16 are submetacentric. The rest are more or less metacentric. Pairs 8 and 9 have secondary constrictions on their short arms.

*Nebrioporus carinatus* (Aubé, 1838), Fig. 3 p (♂), Fig. 3 q (♀), *Nebrioporus fabressei* (Régimbart, 1901), Fig. 3 r, s (♂) and *Nebrioporus croceus* Angus, Fresneda and Fery, 1992, Fig. 3 t, u (♂) were figured and discussed by Angus, Fresneda and Fery (1992) and are shown here for completeness and to permit comparison with the other species.

**The** N. laeviventris **group**

*Nebrioporus amicorum* Toledo, 2009, Fig. 3 v (♂). The RCLs of autosomes 1–19 decrease fairly evenly from about 7.5–3.2. There is then an abrupt decrease to pair 20, RCL about 2, and a further drop to the four smallest pairs, which are more or less dot-like, RCLs 1.5–0.6. The X chromosome metacentric, RCL about 2.7, intermediate in size between autosomes 19 and 20. Autosomes 8, 10, 12, 13, 16, 17 and 20–23 are subacrocentric to acrocentric, and the rest are more or less metacentric. Pair 9 is submetacentric, with a secondary constriction in its long arm.

*Nebrioporus lanceolatus* (Walker, 1871), Fig. 3 w (♂), *Nebrioporus insignis* (Klug, 1833) Fig. 3 x (♂) and *Nebrioporus crotchi* (Preudhomme de Borre, 1871), Fig. 3 y (♂) are Egyptian species and were discussed and figured by Saleh Ahmed et al., 2000. There are figured here, with some slight rearrangement of karyotypes, for completeness and to facilitate comparison with the other species. Apart from the differences among themselves, they differ from *Nebrioporus amicorum* in the smaller number (1 or 2 pairs) of dot-like autosomes.

Nebrioporus **incertae sedis**

*Nebrioporus canariensis* (Bedel, 1881) Fig. 3 z, z’ (♂). The RCLs of autosome pairs 1–23 decrease evenly from about 6.4–2.9. Pair 24 is dot-like, RCL about 1. The X chromosome is submetacentric, RCL about 3.5, comparable with autosome 19. Autosomes 2–4, 10–12, 14, 15, 17, 19 and 21–23 are subacrocentric to acrocentric, and the rest are more on less metacentric.

### StictotarsusZimmermann, 1919

**Figure 4.**
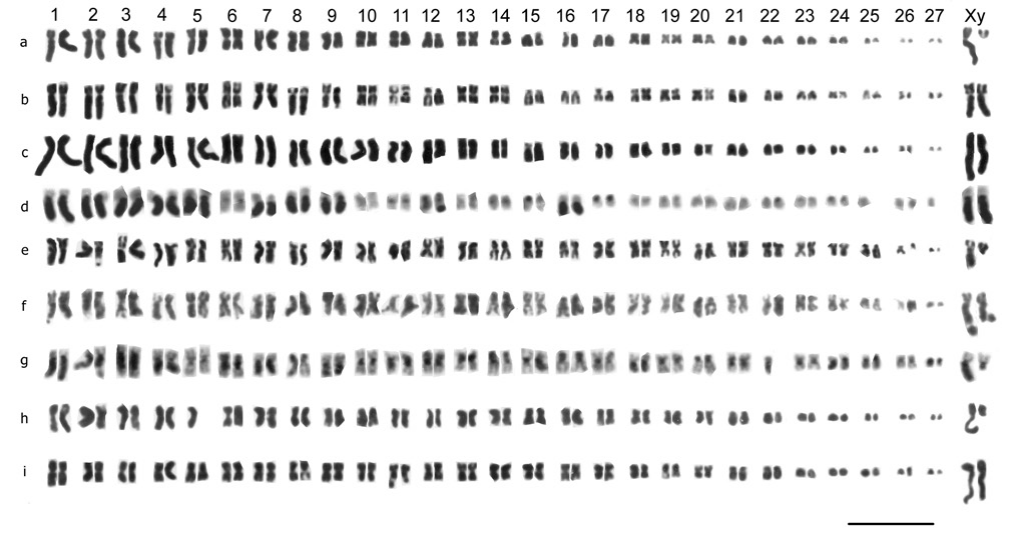
Mitotic chromosomes from mid-gut cells of *Stictotarsus* species, arranged as karyotypes. **a** *Stictotarsus duodecimpustulatus*, ♂, Clatteringshaws Loch, plain; **b, c** *Stictotarsus duodecimpustulatus*, ♀, plain, **b** New Forest **c **Abadia **d** *Stictotarsus duodecimpustulatus*, ♀, C-banded, Esclavitud **e** *Stictotarsus procerus*, ♂, Corsica, Solenzara, plain **f** *Stictotarsus procerus* ♀, Sardinia, Posada, plain **g** *Stictotarsus procerus*, ♂, Corsica, Casaluna, C-banded **h** *Stictotarsus bertrandi*, ♂, pain **i** *Stictotarsus bertrandi*, ♀, plain. Bar = 5µm.

**Figure 5.**
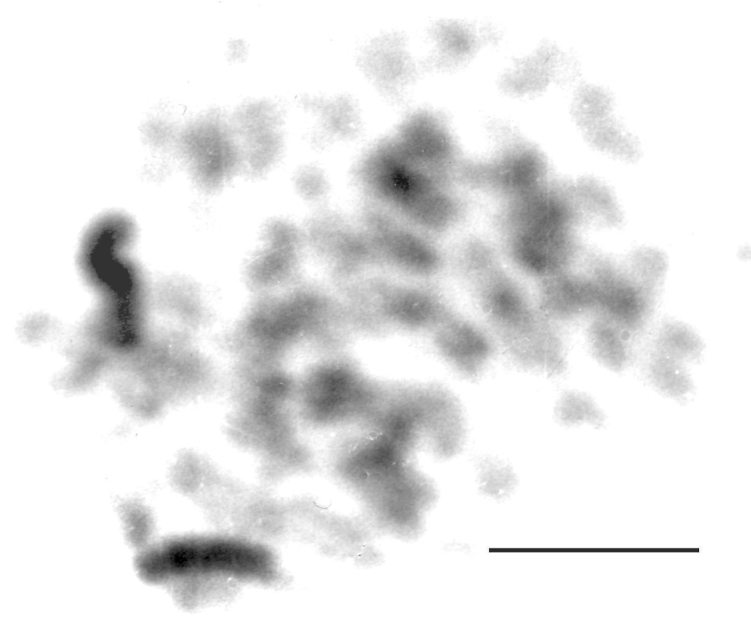
*Stictotarsus bertrandi*, ♀, C-banded nucleus from mid-gut, showing the long heterochromatic arms on the two X chromosomes. Bar = 5µm.

Fig. 4, a – i and Fig. 5 show mitotic chromosomes of the three species studied. All have 27 pairs of autosomes, some of which may be subacrocentric with heterochromatic long arms, X chromosomes with heterochromatic long arms, and a neo-XY system of sex chromosomes, with the Y chromosome resembling the short (euchromatic) arm of the X chromosome.

*Stictotarsus duodecimpustulatus* (Fabricius, 1792). Fig. 4 a (♂), 4 b – d (♀). 2n = 54 + XY (♂), XX (♀). Autosome pairs 1–5, 7–9 and 16 are subacrocentric, and their long arms are entirely heterochromatic (Fig. 4 d). Pairs 1–8 have RCLs ranging from about 7.5–5.7, and this includes pair 6, which is metacentric with a small centromeric C-band. Pair 9, is similar in form to pair 8, but is smaller, RCL about 4.7. Pairs 10–14 show an even decrease in RCL from about 4.2–3.8. There is then an abrupt decrease in RCL with pair 15 having a value of about 3, and pairs 15–21 showing an even decrease in RCL to about 2. The remaining small autosomes show a decrease in RCL from about 1.9–0.9. Pairs 10, 11, 13 and 14 are metacentric with small centromeric C-bands. Pair 12 is subacrocentric and apparently fairly extensively heterochromatic (Fig. 4 d), though without a clear heavily heterochromatic long arm. Pair 17 is acrocentric with a heavy centromeric C-band, and pairs 18–20 are submetacentric with very weak centromeric C-bands. The remaining autosomes are acrocentric, with only pair 22 having a strong centromeric C-band. The X chromosome, RCL about 8, is slightly larger than, and very similar in form to the longest autosomes, and, like them, has a heavily heterochromatic long arm. The y chromosome RCL about 2, is acrocentric and matches the euchromatic short arm of the X chromosome.

*Stictotarsus procerus* (Aubé, 1838). Fig. 4 e, g (♂), Fig. 4 f (♀). 2n = 54 + XY (♂), XX (♀). The karyotype is broadly similar to that of *Stictotarsus duodecimpustulatus*, but with some clear differences. Autosome 1, RCL about 6.2, is subacrocentric with a heavily heterochromatic long arm, but autosome 2 has the heterochromatin apparently confined to the basal half of the long arm (Fig. 4 g).

Autosome 3 is similar in size to pairs 1 and 2, but is metacentric and the middle half of the chromosome is heterochromatic. Autosome 4 is slightly smaller, RCL about 5, but similar in form to pair 1. Autosome 5 is similar in size to pair 4 and is metacentric with a small centromeric C-band, as in *Stictotarsus duodecimpustulatus* autosome 6. Autosomes 6–20 show a steady decrease in RCL from about 5 to about 3.5, with pairs 12–20 all having RCLs of about 3.5. Pair 6 is submetacentric and the short arm is heterochromatic, while pair 7 is subacrocentric with the long arm heterochromatic. Pair 8 is acrocentric without conspicuous C-banding, while pair 9 is almost metacentric with one arm heterochromatic. Pairs 10–13 are metacentric or almost so, with heavy C-bands, which extend onto the shorter arms of pair 12. Pairs 14 and 16 are subacrocentric with heterochromatic short arms. Pairs 15 and 17 are metacentric with centromeric C-bands, and pairs 18 and 19 are metacentric, with a heterochromatic arm in pair 18. Pair 20 is acrocentric with a heavy centromeric C-band. Pairs 21–24 are metacentric, and pair 24 has a heterochromatic arm. Pairs 21 and 22 have RCLs of about 2.6, pairs 23–25 are slightly smaller, RCL about 2.3. Pair 25 is acrocentric with a heavy centromeric C-band. Pairs 26 is smaller again, RCL about 1.3, and is submetacentric with a centromeric C-band, and pair 27 is a small acrocentric, RCL 0.9 or less. The X chromosome, RCL about 5.4 is similar in form to autosome 1. The y chromosome, RCL about 2.2, is acrocentric and matches the euchromatic short arm of the X chromosome.

*Stictotarsus bertrandi* (Legros, 1956). Fig. 4 h (♂), Fig. 4 i (♀), Fig. 5 (♀). 2n = 54 + XY (♂), XX (♀). Although the chromosome number and sex determining mechanism are the same as in *Stictotarsus duodecimpustulatus* and *Stictotarsus procerus*, the general appearance of the karyotype is rather different, with only the X chromosome conspicuously long (RCL about 10.5), subacrocentric and with the long arm heterochromatic. Fig. 5 shows a C-banded preparation of a female mitotic nucleus and although it is not possible to prepare a karyotype from this preparation, the two heterochromatic long arms of the X chromosomes are very distinct. Autosome pairs 1–22 are metacentric or submetacentric, and their RCLs show a gradual decrease from about 6.3 to 3. Autosome 1 is clearly submetacentric, and its longer arm appears to have the chromatids closely applied to one another, suggesting that it is heterochromatic. However, C-banding does not bear this out. It may, however, be the site of a secondary constriction. It may be similar in its structure to the long arm of autosome 2 of *Stictotarsus procerus*, but the material available is not adequate to show this. Autosome pairs 23–27 are acrocentric, and their RCLs range from about 2 to about 1, with pair 27 often appearing dot-like. The Y chromosome, RCL about 2, is acrocentric and matches the short arm of the X chromosome, as is typical of a neo-XY system.

Scarodytes **Gozis, 1914**

**Figure 6.**
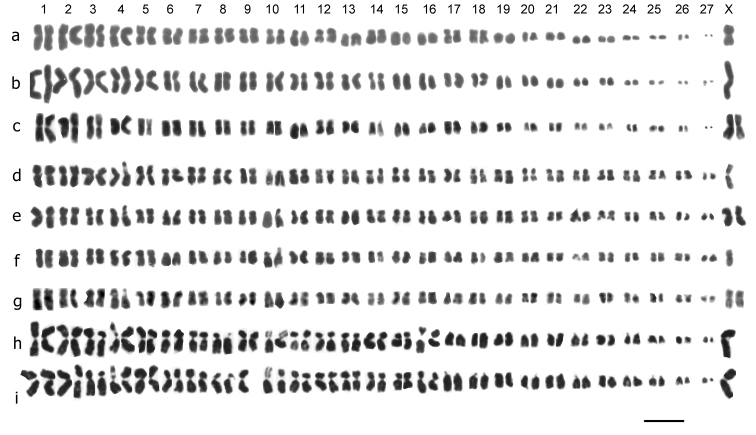
Mitotic chromosomes from mid-gut (**a, c–i**) and testis (**b**) cells of *Scarodytes* species, arranged as karyotypes. **a, b** *Scarodytes halensis*, ♂, Stanton Harcourt **c** *Scarodytes halensis*, ♀, Menton **d** *Scarodytes nigriventris*, ♂, Solenzara **e** *Scarodytes nigriventris*, ♀, Casaluna **f** *Scarodytes fuscitarsis*, ♂, Sardinia **g** *Scarodytes fuscitarsis*, ♀, Sardinia **h, i** *Scarodytes malickyi*, ♂, Crete. Bar = 5µm.

Mitotic chromosomes of the four species analysed are shown in Fig. 6, a – i. All the species have 27 pairs of autosomes and sex chromosomes which are X0 (♂), XX (♀).

*Scarodytes halensis* (Fabricius, 1787). Fig. 6 a, b (♂), Fig. 6 c (♀). 2n = 54 + X0 (♂), XX (♀). The RCLs of the autosomes decrease rather evenly from about 9 to just over 0.5, with pair 27 dot-like. Pairs 1–9, 14, 17 and 18 are metacentric or submetacentric, pairs 10–12 are subacrocentric. Pairs 13, 15, 16 and 19–27 are acrocentric. It is not possible to detect any secondary constrictions in these preparations. The female karyotype, from the French Alpes Maritimes, shows no detectable difference from those of British material.

*Scarodytes nigriventris* (Zimmermann, 1919). Fig. 6 d (♂), Fig. 6 e (♀). 2n = 54 + X0 (♂), XX (♀). The RCLs of the autosomes decrease fairly evenly from about 5.7 to 1.5, and the smallest pair (pair 27) is clearly not dot-like. Pairs 1–3, 5–9, are metacentric to submetacentric, and pair 4 is subacrocentric with a well-marked secondary constriction in its short arm. Pair 10 is acrocentric, 11–14, 17, 18, 24, 25 and 27 are subacrocentric, while the remaining autosomes are submetacentric. The X chromosome, RCL about 6, is submetacentric, similar in appearance to aurosome1. Although *Scarodytes nigriventris* has in the past been placed as a subspecies of *Scarodytes halensis*, its karyotype is clearly different from that of *Scarodytes halensis*, with only about half the range of RCLs, and no dot-like autosomes. This is in complete agreement with the conclusions drawn by Ribera (2003) from his DNA data.

*Scarodytes fuscitarsis* (Aubé, 1838), stat. n. Fig. 6 f (♂), Fig. 6 g (♀). 2n = 54 + X0 (♂), XX (♀). This name is used for Sardinian material which differs from Corsican *Scarodytes nigriventris* in lacking a tooth on the ventral margin of the inner anterior tarsal claw, as well as in having a rather broader body form. This identification has been confirmed by Dr H. Fery (*in litt*. 12.xii.2008). *Scarodytes fuscitarsis*, described from Sardinia, is currently listed as a subspecies of *Scarodytes halensis* (Nilsson, 2001, 2011). *Scarodytes fuscitarsis* is described as having the underside and legs brownish, but in the present material they are black. The general appearance of the karyotype is very similar to that of *Scarodytes nigriventris*, including the secondary constriction on the short arm of autosome 4. It is clearly unlike that of *Scarodytes halensis*, indicating that *Scarodytes fuscitarsis* cannot be a subspecies of it. The range of RCLs is very similar to that shown by *Scarodytes nigriventris*, but the X chromosome appears shorter, RCL about 5.2, and smaller than autosome 1. The morphology of *Scarodytes fuscitarsis* (size and shape of the aedeagus, lack of a tooth on the anterior tarsal claws of the male, size and shape of the beetles) is clearly distinct from *Scarodytes nigriventris*.

*Scarodytes malickyi* Wewalka, 1997. Fig. 6 h, i (♂). 2n = 54 + X0 (♂). The two preparations shown here are difficult to work with because a number of the chromosomes have distinct gaps which appear to be stretched centromeres rather than secondary constrictions (e.g. Fig. 5 h, autosome pair 16. The RCLs of the autosomes range from about 7–0.6, with pairs 17–27 all acrocentric. In both the karyotypes shown here one replicate of autosome 27 is dot-like, but the other clearly is not. More material of this species is needed. Autosomes 1, 2 and 5 are submetacentric, autosome 3 is subacrocentric, while autosome 4, which has the secondary constriction in its short arm, appears submetacentric. Pairs 6–9 are on the border between submetacentric and subacrocentric, and autosome 10 is submetacentric with the short arm appearing rather faint. Autosomes 11–16 are subacrocentric, with pair 16 appearing normal (without the centromeric gap) in Fig. 5 i. The X chromosome is submetacentric, and about as long as autosome 1 (RCL about 6.5).

## Discussion

Two major features emerge from the results presented here: the different species almost all have distinct karyotypes, and within the genera the species tend to have broadly similar karyotypes; and the karyotypes of the different genera are easily reconciled with the phylogram obtained by Ribera (2003, Fig. 3).

In *Deronectes* there is a progressive reduction in the number of autosomes, with an associated increase in their size. In all the species studied the sex chromosome system appears to be neo-Xy.

The karyotypes of *Trichonectes* and *Nebrioporus* agree with one another in having 24 pairs of autosomes and X0/XX sex chromosomes. Relevant interspecies comparisons of *Nebrioporus* chromosomes are given with the results.

The karyotypes of the three *Stictotarsus* species studied here are particularly interesting, not only because of their broad agreement with one another (27 pairs of autosomes and a neo-XY sex chromosome system with the X chromosome having a heterochromatic long arm), but also because the two sibling species (*Stictotarsus duodecimpustulatus* and *Stictotarsus procerus*) have very closely similar karyotypes, while that of *Stictotarsus bertrandi* is more distinct.

The *Stictotarsus* karyotypes all have 27 pairs of autosomes and X0/XX sex chromosomes. Those of Corsican *Stictotarsus nigriventris* and Sardinian *Stictotarsus fuscitarsis* are scarcely different from one another, but the other two species are clearly distinct.

The phylogram given by Ribera (2003) includes all the genera discussed here, plus *Boreonectes* Angus, 2010 and the “*Stictotarsus*” *roffii* group (as “*Stictotarsus griseostriatus*-*roffii* group”) and *Oreodytes* Seidlitz , 1887, with *Laccornis* Gozis, 1914 as an outgroup. This phylogram shows two major groupings. The first comprises two main, well-separated lineages, *Deronectes* and an assemblage including *Boreonectes*, *Oreodytes* and the *“Stictotarsus” roffii* group. The karyotypes of the *Deronectes* species differ from the others mentioned in having a neo-XY sex chromosome system. The karyotypes of the *Boreonectes griseostriatus* species complex are by now well known (Dutton and Angus 2007, Angus 2008, 2010a & b), but those of the other genera remain unknown.

The second major grouping has *Trichonectes* at its base, then branches into a *Stictotarsus* (*duodecimpustulatus* group) plus *Scarodytes* clade and a *Nebrioporus* clade.

The basal position of *Trichonectes* in this second group is very interesting. Its karyotype, with 24 pairs of autosomes plus X0/XX sex chromosome resembles those of all species of *Nebrioporus* for which data are available. This would suggest that the karyotypes of the *Stictotarsus* are highly apomorphic, having evolved not only their own autosome number, but also a common neo-Xy sex chromosome system. In this phylogram *Scarodytes* is placed as sister genus to *Stictotarsus* (*duodecimpustulatus* group), with these two genera as the sister group to *Nebrioporus*. The karyotypes of the four *Scarodytes* species considered here resemble those of *Nebrioporus* in having an X0/XX sex chromosome system, but differ in having three more pairs of autosomes (27 pairs instead of 24).

It can thus be seen that the karyotypes of the genera in this second group could agree with the phylogeny suggested by Ribera. *Trichonectes* would have a karyotype ancestral for the group as a whole, and this would be retained along the *Nebrioporus* clade. However, in the *Stictotarsus* – *Scarodytes* clade two major changes would have occurred; first an increase in the number of autosomes by three pairs (as shown by *Scarodytes*), and second a change in the sex chromosomes to neo-Xy as shown by *Stictotarsus*. Interestingly these *Stictotarsus* resemble *Scarodytes* in the fact that they have 27 pairs of autosomes, but presumably they once had 28 pairs, in order to give an original autosome which could fuse with the original X chromosome to give a neo-X.
